# Complete Genome Sequence of Dehalococcoides mccartyi Strain FL2, a Trichloroethene-Respiring Anaerobe Isolated from Pristine Freshwater Sediment

**DOI:** 10.1128/MRA.00558-19

**Published:** 2019-08-15

**Authors:** Jun Yan, Yi Yang, Xiuying Li, Frank E. Löffler

**Affiliations:** a Key Laboratory of Pollution Ecology and Environmental Engineering, Institute of Applied Ecology, Chinese Academy of Sciences, Shenyang, Liaoning, China; b Department of Microbiology, University of Tennessee, Knoxville, Tennessee, USA; c Center for Environmental Biotechnology, University of Tennessee, Knoxville, Tennessee, USA; d Biosciences Division, Oak Ridge National Laboratory, Oak Ridge, Tennessee, USA; e Joint Institute for Biological Sciences (JIBS), Oak Ridge National Laboratory, Oak Ridge, Tennessee, USA; f Department of Civil and Environmental Engineering, University of Tennessee, Knoxville, Tennessee, USA; g Department of Biosystems Engineering & Soil Science, University of Tennessee, Knoxville, Tennessee, USA; University of Delaware

## Abstract

Dehalococcoides mccartyi strain FL2 couples growth to hydrogen oxidation and reductive dechlorination of trichloroethene and *cis*- and *trans*-1,2-dichloroethenes. Strain FL2 has a 1.42-Mb genome with a G+C content of 47.0% and carries 1,465 protein-coding sequences, including 24 reductive dehalogenase genes.

## ANNOUNCEMENT

Dehalococcoides mccartyi strains are strictly anaerobic, hydrogenotrophic, obligate organohalide-respiring bacteria that conserve energy from the hydrogenolysis of organohalogens (1). D. mccartyi strain FL2 was isolated from Red Cedar River (Okemos, MI, USA) sediment with no history of chlorinated solvent exposure. Strain FL2 shares 99% 16S rRNA gene sequence identity with D. mccartyi isolates of the Pinellas group obtained from contaminated sites (2, 3). The ability of strain FL2 to dechlorinate trichloroethene (TCE) and *cis*- and *trans*-1,2-dichloroethene was attributed to the possession of the TCE reductive dehalogenase (RDase) gene *tceA* (2).

D. mccartyi strain FL2 was grown in defined, anoxic, bicarbonate-buffered mineral salts medium (4, 5) containing acetate as the carbon source. Hydrogen and TCE were provided as the electron donor and acceptor, respectively. Genomic DNA was extracted using the cetyltrimethylammonium bromide method (https://jgi.doe.gov/wp-content/uploads/2014/02/JGI-Bacterial-DNA-isolation-CTAB-Protocol-2012.pdf), and sequencing was performed on a PacBio RS II sequencer (Pacific Biosciences, Menlo Park, CA). DNA shearing (g-TUBE; Covaris, Woburn, MA) generated 8-kb to 10-kb fragments for long-insert library preparation. Hairpin adapters were ligated to fragmented DNA using the SMRTbell template preparation kit (Pacific Biosciences). The BluePippin system (Sage Science, Beverly, MA) was used to size select the final library, which was sequenced in a single-molecule real-time sequencing cell using PacBio P6-C4 chemistry and a 240-minute movie. PacBio raw reads were assembled using the HGAP (SMRT Analysis version 2.3.0; Pacific Biosciences) and Canu (version 1.2) (6) assemblers with default parameters, as described previously (7), and epigenetic base modifications were analyzed using SMRT Analysis 2.3.0 with default parameters. Coding gene prediction and functional annotation of the strain FL2 genome were performed using the NCBI Prokaryotic Genome Annotation Pipeline (8).

The assembled strain FL2 genome comprises one circular chromosome of 1,422,358 bp with a G+C content of 47.0%. A single modified *N*^6^-methyladenosine (m^6^A) base (underlined) was identified at more than 99.6% of the 2,897 GAAGG motif positions in the genome. The genome contains 1,465 predicted protein-coding genes, 46 tRNAs, and single-copy genes for 5S rRNA, 16S rRNA, and 23S rRNA. The 5S rRNA and 23S rRNA genes are colocalized but distant from the 16S rRNA gene. The strain FL2 genome harbors 24 different, single-copy RDase genes, 21 of which are adjacent to a downstream RDase B gene. These 24 RDase genes, including the *tceA* gene, occur in two high-plasticity regions surrounding the origin of replication, a common feature of D. mccartyi genomes (9). A BLASTn search of strain FL2’s RDase genes revealed >99.5% sequence identities to RDase genes reported in other D. mccartyi strains. Genes coding for the catalytic subunit (DhcFL2_00955) and the membrane-bound subunit (DhcFL2_00950) of a complex iron-sulfur molybdoenzyme (CISM) (10) and the large subunit (DhcFL2_00385) and the small subunit (DhcFL2_00390) of a Ni-Fe hydrogen uptake hydrogenase (Hup) (10) were present in single copies. A 48.3-kb prophage region (positions 858640 to 906950) was identified and annotated using PHAST (11). The majority of the 27 phage-related genetic elements code for hypothetical phage (structural) proteins, which were assigned by BLASTp hits to a diversity of bacteriophages. The strain FL2 genome expands the D. mccartyi pangenome and provides information for comparative genomic studies.

### Data availability.

The complete genome sequence of Dehalococcoides mccartyi strain FL2 has been deposited in DDBJ/ENA/GenBank under the accession number CP038470. The BioSample and BioProject accession numbers are SAMN11289547 and PRJNA529963, respectively. Raw sequences have been deposited in the Sequence Read Archive (SRA) under the accession number SRR9599543.
